# *Saccharomyces cerevisiae* and its industrial applications

**DOI:** 10.3934/microbiol.2020001

**Published:** 2020-02-11

**Authors:** Maria Parapouli, Anastasios Vasileiadis, Amalia-Sofia Afendra, Efstathios Hatziloukas

**Affiliations:** 1 Molecular Biology Laboratory, Department of Biological applications and Technology, University of Ioannina, Ioannina, Greece; 2 Genetics Laboratory, Department of Biological Applications and Technology, University of Ioannina, Ioannina, Greece

**Keywords:** *Saccharomyces cerevisiae*, non-*Saccharomyces* yeast, wine yeast, Baker's yeast, cocoa fermentation, bioethanol

## Abstract

*Saccharomyces cerevisiae* is the best studied eukaryote and a valuable tool for most aspects of basic research on eukaryotic organisms. This is due to its unicellular nature, which often simplifies matters, offering the combination of the facts that nearly all biological functions found in eukaryotes are also present and well conserved in *S*. *cerevisiae*. In addition, it is also easily amenable to genetic manipulation. Moreover, unlike other model organisms, *S*. *cerevisiae* is concomitantly of great importance for various biotechnological applications, some of which date back to several thousands of years. *S*. *cerevisiae*'s biotechnological usefulness resides in its unique biological characteristics, i.e., its fermentation capacity, accompanied by the production of alcohol and CO_2_ and its resilience to adverse conditions of osmolarity and low pH. Among the most prominent applications involving the use of *S*. *cerevisiae* are the ones in food, beverage -especially wine- and biofuel production industries. This review focuses exactly on the function of *S*. *cerevisiae* in these applications, alone or in conjunction with other useful microorganisms involved in these processes. Furthermore, various aspects of the potential of the reservoir of wild, environmental, *S*. *cerevisiae* isolates are examined under the perspective of their use for such applications.

## 1. Introduction

*Saccharomyces cerevisiae* (*S. cerevisiae*) is a unicellular fungus, possessing a nuclear genomic DNA of 12068 kilobases (kb) organized in 16 chromosomes [1]. Its genome has been completely sequenced by Goffeau et al. 1996 [1] and was found to contain approximately 6000 genes, of which, 5570 [2] are predicted to be protein-encoding genes. Bioinformatic analyses have revealed that a number of protein-encoding genes are of foreign origin, i.e., a result of lateral gene transfer, as the term was defined by Doolittle, 1999 [3]. These genes, which entered *S. cerevisiae*'s genome horizontally, are either of prokaryotic or eukaryotic origin [4]. This came initially as a surprise, because of its osmotrophic nutritional style and the presence of robust cell wall, cell- and intracellular membranes. Hall at al., 2005 [4] located 10 genes of putative prokaryotic origin present in *S. cerevisiae*'s genome. One example of acquisition of a gene from another eukaryote is the gene *FSY1*. *FSY1* encodes a fructose transporter [5] and has probably originated from some close relative of *S. cerevisiae*. This gene is considered as important because its product lends probably to its host strain (EC 1118) an increased capability to utilize fructose under conditions of low hexose concentrations present in the must (i.e., towards the end phase of fermentation).

In respect to *S*. *cerevisiae* extra chromosomal elements' genomics, all strains contain of course mitochondrial DNA (mtDNA) molecules, but often with different sizes [6]. The largest version of mtDNA has a length of approximately 85780 bps [7]. Furthermore, most *S. cerevisiae* strains harbor in their nucleus a distinct extra-chromosomal DNA genetic element called 2µm circle (reviewed by Futcher, 1988 [8]). This double-stranded DNA element has a typical length of 6318 bps and a copy number of approximately 60 copies per cell). It is considered as ‘selfish DNA’ and has nearly no phenotypic consequences for its host, except a slight reduction of the host's growth rate. It is of no use for industrial applications, but on the other hand was highly instrumental for various applications concerning the genetic manipulation of its host. Other extra-chromosomal genetic elements harbored by various strains of *S. cerevisiae* include single- and double-stranded RNA molecules and retroviruses [9]. Some of these elements have a significant contribution to *S*. *cerevisiae*'s killer phenotype (s. Section 2.2.2).

*S. cerevisiae* is a model organism, a valuable tool for all aspects of basic research. Unlike other model organisms though, such as *Escherichia coli*, or *Caenorhabditis elegans*, *S. cerevisiae* is concomitantly also a most valuable species for a variety of industrial applications. One major reason for this feature is one part of its life style, termed ‘make-accumulate-consume’ [10]. This feature is based on the Crabtree effect, which consists in the fact that *S. cerevisiae*, even under aerobic conditions does not use the respiratory machinery to metabolise saccharides and promote biomass growth, but instead, it produces ethanol and other two-carbon compounds, via pyruvate [11]. The consequence of this fact is that *S. cerevisiae* produces and accumulates ethanol—which is toxic, or static, for most other microbial species able to compete with it for the sugar compounds- and thus eliminate competition. After *S. cerevisiae* has cleared the particular ecological niche from most of its competitors, it then proceeds in the consumption of the produced ethanol, thus promoting its own growth. According to Hagman et al., 2013 [12], this strategy evolved gradually before the whole genome duplication of *S. cerevisiae* and other yeast species, which took place approximately 100 million years ago [13]. It consisted in the loss of a specific cis-acting regulatory sequence (AATTTT) of several promoters, of genes involved in respiration [14]. This sequence is present and conserved in many other yeast genera, such as, *Kluyveromyces*, *Candida* and others, while it is absent from the yeast *Dekkera*, a genus, which includes species, known to be efficient ethanol producers [15]. Certainly, there are two more characteristics, which are very important for some industrial applications of *S. cerevisiae*: its remarkable resistance/tolerance to high sugar concentrations and production of a number of aromatic, volatile compounds. To the latter characteristic will be devoted special attention during the discussion of vinification.

Environmental strains of *S. cerevisiae* are subjected to much harsher conditions, than the laboratory ones, which are usually cultured under most favourable conditions. The study of environmental strains reveals, among others, also additional survival strategies developed by this species, which are not apparent during the studies of laboratory/industrial strains. Environmental strains are able to overwinter in the soil, where they can sporulate. Other known natural niches, which *S. cerevisiae* usually occupies, are leaves and trunks of various plant species, such as oak trees. It is noteworthy, that although *S. cerevisiae* is found in abundance in environments, such as wineries, its presence there does not originate from grapevines, or grape berries. To the contrary, its presence in the latter habitats is scarce compared to other microorganisms. Mortime and Polsinelli, 1999 [16] determined the frequency of *S. cerevisiae*'s presence in one in a thousand grapes, a frequency much smaller than the ones of other microorganisms. In addition, they found that the incidence of *S. cerevisiae* increases to one out of four when it concerns damaged grapes in the field. In a different study, Taylor et al., 2014 [17], using a metagenomic approach, were able to detect approximately one *S. cerevisiae* cell among approximately 20,000 cells belonging to various other fungal genera/species. The rare presence of *S. cerevisiae* in intact grapes and its much frequenter presence in damaged ones seem to constitute a contradiction, which is explained though by the fact that this organism can occupy an additional niche, i.e., insects. *S. cerevisiae* is insect-borne, and was detected in several different insects, such as, wasps [18] and *Drosophila* species [19], which feed on, among others, also on damaged grapes. Stefanini et al., 2012 [18] examined the gut microbiome of social wasps and detect the presence of *S. cerevisiae* cells, albeit in smaller numbers (4%) compared with other yeasts, such as *Candida*, or *Pichia*. Despite its smaller numbers, *S. cerevisiae* has a stable presence in the wasp community, since it overwinters in the gut of hibernating colony founding queens from autumn until spring and then, is transferred to their larvae through feeding. Regarding the *Drosophila* - *S. cerevisiae* interaction, Buser et al., 2014 [19] in the course of their study of the niche construction theory, showed that *Drosophila* *simulans* has a preference for yeast producing more efficient attractants. This is a mutually beneficial interaction, because while the flies that harbor the yeast exhibit an increased fecundity, *S. cerevisiae* benefits from being transferred to new niches, such as damaged grapes. This explains the higher incidence of *S. cerevisiae* in damaged grapes, compared with the incidence on intact ones. The frequency of *S. cerevisiae* occurrence in the environment is still under study, but it is much frequenter than initially anticipated. Wang et al., 2012 [20] collected 2064 samples from various natural, not human-made, habitats in China and were able, using an enrichment-based approach, to detect the presence of *S. cerevisiae* in 226 of them (10.9 %). The genetic diversity of *S. cerevisiae* isolates found in the positive samples was also much larger than in human-made, or human–‘influenced’ ones. This could be potentially very important, because environmental, ‘wild’, strains could bear genotypes with highly interesting properties for biotechnological applications. The use of ‘wild’ strains for industrial applications though may not be a simple procedure, because the genetic diversity does not always correspond to a phenotypic one. Camarasa et al., 2011 [21] e.g., studied the efficiency of 72 *S. cerevisiae* strains of diverse origins (industrial, laboratory, environmental) under conditions of must fermentation and found that strains originating from rich in sugar environments were able to finish the fermentation process, while the laboratory or environmental strains were unable to perform satisfactorily. The molecular basis of the better adaptation, especially of the wine strains, to the stressful conditions of must fermentation is yet unknown and could be attributed more than one reasons such as epigenetic phenomena.

This review focuses mainly on various aspects of industrial applications employing *S. cerevisiae*, especially those related to its fermentation capacity and its use in the wine and food industry, as well as in the bioethanol production. Furthermore, we present data highlighting the potential of environmental *S. cerevisiae* isolates in the above-mentioned biotechnological applications.

## 2. Application of *S*. *cerevisiae* in the beverage and food industry

*S*. *cerevisiae* has been an essential component of human civilization because of its extensive use in food and beverage fermentation in which it has a high commercial significance. In the European yeast industry, a 1 million tonnes is produced annually, and around 30% of which is exported globally. The global market's annual growth rate was 8.8% from 2013 to 2018.

In regard to beverage industry, *S. cerevisiae* is involved in the production of many fermented beverages, such as wine, beer and cider; distilled beverages, such as rum, vodka, whisky, brandy, and sake; whereas in other alcoholic beverages worldwide, from fruits, honey, and tea, *S. cerevisiae* is also involved [22]. Fermentation can take place either from a spontaneous development of the raw material microflora, or from the addition of a pure yeast culture. [22],[23]. A discussion on the contribution of *S. cerevisiae* in wine, bread and cocoa fermentations follows, highlighting aspects such as the biochemical reactions that take place in the cell and whose products determine the final products, the traits that strains must have in order to be successful starters and the potential of exploiting native strains in industry.

### 2.1. Application of *S. cerevisiae* in the wine industry

The relationship between wine and man dates back thousands of years: detection of calcium salt of tartaric acid and terebinth resin in a pottery jar constitute the first experimental evidence for the presence of wine in Iran, around 5400–5000 BC [24]. The relationship between wine and *S. cerevisiae* is equally long-lasting as it was proven by the presence of ribosomal DNA from *S. cerevisiae* in a wine jar from Egypt dated back to 3150 BC [25]. However, this relationship was revealed not earlier than 1860 when Louis Pasteur established for the first time the ‘hidden’ world of yeast activity during the wine fermentation [26] and eventually in 1890, when Müller-Thurgau proposed the process of controlled wine fermentations with starter cultures [27]. This innovative practice, which found broad application after almost a century, in the 1970s, revolutionized the wine industry and has resulted in the improvement of wine quality by offering a better control and consequently better repeatability and reliability of the fermentations [27].

From the very beginning, the vinification environment exposes microorganisms present in grape must to many different types of stress applying selective pressure on them [27]–[30]: Natural grape must is hostile due to its low pH and high sugar concentrations; in the majority of industrial fermentations high concentrations of the antioxidant and antimicrobial preservative sulphur dioxide are also added intensifying harsh conditions, whereas, as the fermentation proceeds, stress is multiplied for a plethora of reasons including anaerobic conditions, depletion of nutrient reserves (nitrogen, lipids and vitamins), increased acid concentrations, ethanol toxicity and temperature variations. *S. cerevisiae* although is found in very low populations in vineyards or grapes, prevails fermentation by dominating over the other yeast species abundant in natural must as it is able to overcome all fermentation stresses [28],[31]. This is the reason it has gained itself the title of ‘the wine yeast’ being the main workhorse of the wine industry worldwide [31]. Another set of criteria for a successful choice of a starter culture in industry is the production levels of several metabolites that, form the ‘fermentation bouquet’ and determine the complex sensorial and organoleptic character of the produced wine [29]. As it has been reported the compounds that have the greatest impact on ‘fermentation bouquet’ include higher alcohols, esters, aldehydes and terpenes [32]. *S. cerevisiae* is primarily responsible for the formation of the first three categories as it is not a sufficient terpenes producer [33].

Higher alcohols (fusel alcohols), from a quantitative point of view, is the most important group of compounds that *S. cerevisiae* produces during fermentation. Their biosynthesis is conducted via amino acid catabolism, through a route known as the Ehrlich pathway [34]. As it is reviewed by Hazelwood et al., 2008 [35] this pathway consists of three steps: Initially transaminases, encoded by the genes *ARO8*, *ARO9*, *BAT1* and *BAT2*, deaminate amino acids to the corresponding α-ketoacids. Secondly, α-ketoacids are converted to their corresponding aldehydes by one of the five decarboxylases (Pdc1p, Pdc5p, Pdc6p, Aro10p and Thi3p) present in *S. cerevisiae* genome. Finally, the alcohol dehydrogenases, Adh1p to Adh6p and Sfa1p, catalyze the reduction of aldehydes to their corresponding higher alcohols. Styger et al., 2011, 2013 [36],[37] exploited the yeast deletion library EUROSCARF in order to access the genes that have the most important contribution on higher alcohol production. Their results highlight *BAT2* as the dominant gene of the pathway suggesting that the initial transamination step is rate-limiting. Typical representatives of higher alcohols found in wine are 1-propanol (stupefying), 1-butanol (fusel odor), isobutanol (alcoholic flavour), 2 phenylethanol (floral, rose notes) and isoamyl alcohol (marzipan flavours) [38]. Because if their total concentration exceeds 400 mg/lt, they contribute negatively on the wine bouquet [38], industry demands for strains with a relatively low fusel alcohol production [29]). The correlation between the concentration of amino acids and the amount of higher alcohols produced follows a pattern according to which at YAN (Yeast Assimilable Nitrogen) levels below 200 mg/L, the production of higher alcohols increases along with YAN concentrations whereas above 200 mg/L the relationship turns inversible [39],[40], a trend that should be taken into account to modulate higher alcohols formation in industry. The concentrations that are reached however are strongly related to the strain used [41].

Esters are the most desirable group of compounds contributing fruity and floral aromas to the wine bouquet [42]. *S. cerevisiae* synthesizes two major groups of esters during fermentation, namely the acetate esters of higher alcohols and the ethyl esters of medium-chain fatty acids [MCFA]. Such esters include ethyl acetate (varnish, nail polish, fruity), isoamyl acetate (banana, pear), isobutyl acetate (banana) and phenylethyl acetate (rose, honey, fruity, flowery), ethyl hexanoate (apple, banana, violets), ethyl octanoate (pineapple, pear) and ethyl decanoate (floral) [43]. Recently, Ruiz et al. 2019 [44] reviewed extensively the correlation of the most important volatile compounds in wines with their corresponding odor in many different vine varieties. At this point it should be noted that ethyl acetate is desirable at concentrations below 150 mg/L, otherwise it confers spoilage character to wine [43]. Because the concentration of most esters is low and therefore very close to the human's smell nose detection limit, we understand that minimal variations in their concentration can to be of great importance for the quality of the final product of fermentation (wine) [30],[45]. The biosynthesis of acetate esters takes place intracellularly through an enzymatic reaction between acetyl-coenzyme A and an alcohol catalyzed by an alcohol acetyltransferase (AATase). In *S. cerevisiae*, two such enzymes have been extensively studied: AATase I and AATase II encoded by genes *ATF1* and *ATF2*, respectively [42]–[48], whereas recently an ethanol acetyltransferase, encoded by *EAT1*, was also identified [49] and reported to have the potential to produce acetate and propanoate esters [50]. Eht1p and Eeb1p are the enzymes that catalyze the reaction between a medium chain fatty acid [MCFA]-CoA with ethanol synthesizing the MCFA-ethyl esters, possessing also as well as an esterase activity [51]. With regard to acetate ester hydrolysis, the only enzyme identified in the *S. cerevisiae* proteome is isoamyl acetate-hydrolyzing esterase [Iah1p] [48],[52],[53]. Experiments by Kruis et al., 2018 [50] revealed that even when all known ester synthases were deleted in *S. cerevisiae*, ester biosynthesis was not completely abolished, suggesting the existence of other ester synthases in the *S. cerevisiae* proteome, which have not been discovered yet. In addition, the determination of esterase activity in partially purified protein fragments of *S. cerevisiae* cells leads to the conclusion that other unidentified enzymes with esterase activity are present in the *S. cerevisiae* proteome [42]. Based on the needs of modern wine making, successful starters should possess moderate esterase activity [29].

The major aldehyde synthesized by *S. cerevisiae* during wine fermentation is acetaldehyde constituting over 90% of the total aldehyde content of wine [54],[55]. Acetaldehyde, is the last precursor in the anaerobic pathway before ethanol. The pyruvate, end product of glycolysis, is converted to acetaldehyde by the pyruvate decarboxylase enzymes, encoded by genes *PDC1*, *PDC5*, and *PDC6* with the first two pdc enzymes, being the major contributors to the decarboxylation activity controlling directly levels of acetaldehyde [56]. Acetaldehyde is further converted to ethanol, by the *ADH1*-encoded dehydrogenase, whereas *ADH2*-encoded enzyme [Adh2p] catalyzes the reverse reaction. Acetaldehyde when present in low concentrations confers a fruity pleasant aroma but when in excess produces green and grassy off-flavours [54]. Worth of notice though is that the concentrations of acetaldehyde vary significantly between different types of wine with average values of approximately 80 mg/L for white wine, 30 mg/L for red wine, and 300 mg/L for sherries [54]. In addition to the direct effect of acetaldehyde on the aromatic profile of wine, it is equally important, if not more, its indirect effect due to its high reactivity with other compounds [36],[54]. Of special interest for the wine industry it is acetaldehyde's binding activity with SO_2_, the basic antimicrobial and antioxidant agent, forming a complex compound which offers limited protection to the produced wine [54]. Acetaldehyde also mediates condensation reactions of grape-derived anthocyanins with tannins into stable red wine pigments during winemaking [57]. As different strains of *S. cerevisiae* synthesize considerably different amounts of acetaldehyde, it is critical to choose a strain suitable depending on the type of wine produced [54],[58],[59].

The process of producing the fermentation bouquet is complex. It includes a significant number of biosynthetic pathways and genes and is affected by various parameters including the composition of the fermentation medium, the fermentation conditions and the inoculum used [32]. Although genetic manipulation of *S. cerevisiae* strains has already been proposed since the beginning of the century in order to generate strains with an ideal combination of desired oenological traits [60], however, as practice proves, it is not an alternative that finds ground in industry that prefers natural strains to cover consumers' demands. Many studies have shown that the use of starter cultures consisting of indigenous to the winemaking environment strains enhance wine flavour and confer to wine a special distinctive character reflecting the area of origin (terroir) [61]–[65]. However, in industry spontaneous fermentations involving indigenous microflora are not carried out due to their very serious disadvantages including inconsistent results from vintage to vintage and prevailing of undesired microorganisms resulting in the production of off-flavours spoilage of wine [66]. Instead, winemaking in industry worldwide is usually carried out with the use of a limited number (<150) of commercially available *S. cerevisiae* strains [60]. Although this practice ensures reliability and reproducibility, however, it leads to the production of ‘industrialized’ wines in which the positive influence of indigenous flora has been minimized [62]. Given the fact that the microflora of the vineyards is characteristic, demonstrating the existence of a nonrandom microbial-terroir, depending on regional, varietal and climatic factors, it is easily concluded that there is a rich source of indigenous *S. cerevisiae* strains to be exploited by the wine industry [67]. To this end, this practice can guarantee the preserving of the microbial biodiversity and the improvement of product quality leading to better consumer acceptance and consequently higher economic profit of the wine industries.

### 2.2. Alternative vinifications employing mixed yeast inocula of *S. cerevisiae* and non-*Saccharomyces* species as starters

In recent years, several researchers and many wine industries have turned their attention to vinification processes involving fermentations with mixed yeast inocula. There are two main reasons for this approach: i) the attempt to improve the organoleptic characteristics of the resulting wines. ii) The need for production of wines with lower alcohol content.

The mixed starter cultures usually consist of one commercial, or laboratory *S.* *cerevisiae* strain and one non-*Saccharomyces* strain, usually isolated from grapes. As non-*Saccharomyces* inocula have served member species of several genera, among them *Candida*, *Debaryomyces Hanseniaspora* (and its anamorph *Kloekera*), *Issatchenkia*, *Kluyveromyces*/*Lanchancea*, *Metschnikowia*, *Pichia*, *Torulaspora*, *Wickerhamomyces* (Table 1). The mixed starters are applied in a co-, or sequential fashion and often in varying ratios of cell numbers.

#### 2.2.1. Improvement of the organoleptic characteristics of the wine

The use of non-*Saccharomyces* species as starters, along with various *S*. *cerevisiae* strains improved considerably various wine characteristics, such as: physicochemical properties, the composition and concentration of the wine's volatile compounds, i.e. flavour, aroma of the final product, glycerol concentration and others. Ciani et al., 2016 [68] reviewed such efforts published up to 2009. In Table 1, are presented some representative examples and its achievements, or failures of similar mixed starter efforts after 2009.

#### 2.2.2. Reduction of the ethanol content of wines

The efforts to lower the ethanol content of wines emanate from two reasons: the consumers' desire for wines with less alcohol and the climatic change and global temperature increase, which, among others, leads also to higher sugar content of the grapes and subsequently to higher ethanol content in wines. Furthermore, wines with lower alcohol content are subject to lower taxes and therefore, it has a positive effect on the product's final price.

Aiming the reduction of alcohol content of wines, a number of approaches were developed. There are three categories of approaches: application before, during and post fermentation.

##### 2.2.2.1. Approaches applied before fermentation begin

One approach is the selection of varieties, or clones bearing grapes with lower sugar content, or the earlier harvest of the grapes, before they accumulate sugar. Early harvest though has a negative impact on the maturity of important phenolic compounds and the final aromatic profile of the wine [82]. Furthermore, various cultivation practices were explored aiming at the production of grapes with lower sugar content [83], but they often proved ineffective.

Physical methods were also developed and applied prior to fermentation begin. They include various membrane nanofiltration methods to remove glucose from the must (indicative references: [84],[85]. For the same purpose was used also reverse osmosis, resulting in altered organoleptic and physical characteristics [86]. Another possible approach is the treatment of must with the enzyme glucose oxidase, which oxidizes glucose to prior to fermentation start, which though leads also to the unwanted oxidation of other compounds as well [83].

##### 2.2.2.2. Approaches applied during fermentation

Decrease of the alcohol content during fermentation was attempted using genetically modified *S. cerevisiae* with partial success, or an alternative approach based on adaptive evolution [83],[87]. However, the most widely used approach by researchers and wineries and probably the most inexpensive and efficient one is the employment of mixed starters, composed of *S*. *cerevisiae* and some other yeast species. In one occasion, *S. cerevisiae* was combined with another *Saccharomyces* species, *Saccharomyces* *kudriavzevii* [88]. The authors used different aeration conditions and ratios of S. *cerevisiae*–*S*. *kudriavzevii* inocula, achieving under specific conditions a reduction of ethanol concentration up to 1.9 %. In most cases, the mixed inocula consist of one *S.* *cerevisiae* and one non-*Saccharomyces* species. The various combinations used for the purpose of reducing the alcohol content in wines are presented by Ciani et al., 2016 [68].

**Table 1.** Mixed starter cultures of *S*. *cerevisiae* with non-*Saccharomyces* species leading to improved organoleptic traits.

Inocula composition	Fermented material	Mode of inoculation	Major achievement/negative results	Reference
*S. cerevisiae–Candida sake*	Grape must	Co-inoculation	high levels of esters and fatty acids enzymatic activities	Maturano et al., 2015 [69]
*S. cerevisiae–Debaryomyces vanrijiae*	Grape must	Co-inoculation	Increased levels of terpenes and higher alcohols	Maturano et al., 2015 [69]
*S. cerevisiae–Hanseniaspora uvarum*	Grape must	Co- and sequential inoculation	Increased production of volatile compounds, esters and terpenes	Tristezza et al., 2016 [70]
*S. cerevisiae–Hanseniaspora vineae*		Co- and sequential inoculation	Two-fold increase of the concentration of 2-phenylethyl acetate in the sequential inoculation	Viana et al., 2011 [71]
*S. cerevisiae–Hanseniaspora vineae*	Grape must	Sequential inoculation	Increased production of acetate esters and some ethyl esters, decreased production of higher alcohols and some medium chain fatty acids	Medina et al., 2013 [72]
*S. cerevisiae–Issatchenkia orientalis*	Grape must	Co-inoculation	Reduction of malic acid	Kim et al., 2008 [73]
*S. cerevisiae–Lanchancea thermotolerance*	Grape must	Co- and sequential inoculation	Reduction of pH, increase of 2-phenylethanol and glycerol	Gobbi et al., 2013 [74]
*S. cerevisiae–Metschnikowia pulcherrima*	Grape must	Co-inoculation	Increased production of polysaccharides, glycerol and volatile compounds. Reduction of volatile acidity.	Comitini et al., 2011 [75]
*S. cerevisiae–Metschnikowia pulcherrima*	Mango pulp	Co-inoculation	Increased glycerol concentration, reduction of volatile acidity and total acidity	Sadineni et al., 2012 [76]
*S. cerevisiae–Metschnikowia pulcherrima* var. *zitsae*	Grape must	Sequential inoculation	Improved aromatic bouquet.	Parapouli et al., 2010 [77]
*S. cerevisiae–Pichia guilliermondii*	Grape must	Sequential inoculation	(Negative outcome) Production of taste spoiling phenol compounds	Sáez et al., 2010 [78]
*S. cerevisiae – Torulaspora delbruckii*	Amarone must		Improved aroma	Azzolini et al., 2012 [79]
*S. cerevisiae – Torulaspora delbruckii*	Grape must	Co-inculation	Increase of ester production	Renault et al., 2015 [80]
*S. cerevisiae – Torulaspora delbruckii*	Mango pulp	Co-inoculation	Increased glycerol concentration, reduction of volatile acidity and total acidity	Sadineni et al., 2012[76]
*S cerevisiae – Wickerhamomyces anomalus*	Grape must	Sequential inoculation	Increased levels acetates, ethyl esters and lineal alcohols. Reduced levels of organic acids	Izquierdo- Canas et al., 2014 [81]

Yeast-yeast interactions during fermentation: Despite the reported successes achieved using mixed inocula of *S.* *cerevisiae* and non-*Saccharomyces* species, the choice of combination is not straight forward. The reason is that the interaction among different yeast species is not only species but also strain specific [89]. For example, during a fermentation employing *S*. *cerevisiae* and *T*. *debrukii*, Curiel et al., 2017 [90], found that several genes (44) were up-regulated at the early stage of fermentation (2 h). Among these genes were genes involved in the ‘Glucose Fermentation Pathway’, many genes whose products are involved in the alternative nitrogen assimilation pathway, as well as several genes encoding amino acid permeases. In the case of a similar fermentation involving *S*. *cerevisiae* and *C*. *sake*, Curiel et al., 2017 [90] found many similar genes up-regulated, as in the case of *T*. *debrukii*, while in the interaction with *C*. *sake*, 34 genes were down regulated, unlike the case of *T*. *debrukii*, where only four genes were down-regulated. Markedly different was the gene regulation profile of *S*. *cerevisiae* when combined *H*. *uvarum* in a similar fermentation. In this case, Curiel et al., 2017 [90] found that a set of 29 genes were up-regulated, among them also genes involved in stress response.

In general, there is a broad spectrum of different kinds of interactions between must fermenting species/strains. This spectrum ranges from the production of compounds lethal or static to other yeast and bacterial species/strains up to neutral coexistence, or even to mutualism. Branco et al., 2014 [91], e.g., identified several antimicrobial peptides secreted by *S*. *cerevisiae* CCMI 885, which had either a lethal or a static effect on other yeast species and *Oenococcus* *oeni* strains during fermentation in synthetic must. These peptides are fragments derived from the enzyme glyceraldehyde-3-phosphate dehydrogenase. In addition, several *S*. *cerevisiae* strains secrete proteinaceous compounds named K1, K2 and K28, which kill other, sensitive *S*. *cerevisiae* strains. These toxin-like substances are products of genes present in extra-chromosomal RNA genetic elements [9]. Other types of yeast-yeast interactions include cell-to-cell contact mediated domination [92], mildly antagonistic coexistence during fermentation, such as those presented in Table 1, or even stimulation of metabolic activity [90]. Therefore, although each new combination of starters needs to be tested carefully, it seems that there are a vast number of possible new combinations of mixed starters.

##### 2.2.2.3. Approaches applied after the fermentation

All these approaches for ethanol removal post fermentation from wine are based on various membrane filtration techniques, reverse osmosis and evaporation of alcohol. This approach though impacts negatively and significantly the profile of volatile compounds, especially various esters [93].

### 2.3. Applications of *S. cerevisiae* in the bread industry

#### 2.3.1. *S. cerevisiae* in bread

The practice of bread making is one of the oldest biochemistry processes in the world. There are strong indications that yeast was already used in 10.000 BCE to produce bread but the earliest archaeological evidence for leavened breads was found in the second millennium BC in Egypt and the first millennium BC in North Western China. Until the middle ages bread was mostly made at home, but during the population expansion of the 11th and 12th centuries, communal mills and ovens were constructed and professional bakers became common [94]–[98].

*S*. *cerevisiae*, also known as baker's yeast or simply ‘the yeast’, is the most common yeast species in bread and in sourdoughs. It has been used as a starter culture since the 19th century, where the Baker's yeasts were obtained from the leftovers of the beer manufacture. In 1792 in England, the first compressed yeasts for baking and brewing were made and by 1800 they were available in northern Europe, while in the U.S.A. in 1868, a compressed yeast of an improved strain was introduced and facilitated the large-scale production of bread [94]–[100].

Bread production requires the mixing of flour, water and sourdough. Depending on the culture and geographic location, different kinds of flours were used including wheat, barley, emmer, einkorn, khorasan, rye, spelt, teff, maize, or sorghum, while the sourdough was a mix of flour and water, containing fermenting yeast and Lactic Acid Bacteria (LAB) [98]. *S. cerevisiae* is generally inoculated into bread dough at a concentration of 2% of the total ingredients. The oxygen from the air entrapped in the dough during mixing is consumed in a couple of minutes by the respiration of yeast cells, and under the anaerobic conditions that are formed yeast cell reproduction is slowing down and the fermentation reaction takes place. The optimal conditions for fermentation in the dough are around 34–38 °C, at pH 4.0–5.2, using fresh cells because older cells require longer fermentation time. A possible factor that delays yeast multiplication is the addition of fat, salt, or spices [101].

Three categories of sugar exist on dough: a) natural sugars present in flour (including glucose, sucrose, fructose, and maltose), b) added sugars by bakers, and c) maltose released by the amylolytic breakdown of starch. The yeast cells transform glucose and fructose from the degradation of the more complex carbohydrates such as sucrose, maltose, and starch, to carbon dioxide and ethanol. Maltose, dextrose and sucrose are produced from the starch with the help of amylases found in the flour or in diastased malt, besides the possible addition of fungal amylases added by bakers, while glucose and fructose are transformed into carbon dioxide and ethanol by zymases [22],[94],[98],[101].

Because of the sugar fermentation, the yeast cells are considered as a leavening agent in baked goods, leading to an increase of the bread dough volume from the fermentation gasses, therefore to changes in the structure of the product, and to synthesis of organic acids and volatile products that contribute to the taste and flavour of bread [94],[101]. When the most favorable environment for yeast growth is provided, the yeast fermentation produces gases and forms a gluten matrix that enables maximum gas retention, thus achieving a desired loaf volume. The incorporated into the matrix gas bubbles are growing during fermentation, and are getting saturated with carbon dioxide, leading to the expanding of the dough and thinning of the dough matrix between the gas cells. The gas holding capacity of the dough is an important characteristic that determines the bread quality and suitability of the yeast in use. More gas cells in the dough, the higher the gas cells distribution is, leading to more resistance in forces that may cause them to rupture, to lower extensibility and to a higher specific volume. During baking, the ethanol from the dough evaporates along with some water forming the aerated matrix of the crumb [94].

With the use of dried yeast, nonviable cells are present, releasing glutathione as a stress response, while instant active dry yeast helps reduce dough mixing time, due to an effect on the gluten network development. Dough is affected by oxidizing and reducing agents, such as glutathione, which affects the disulfide bonds on the glutenin subunits and their degree of polymerization, resulting in a modified viscoelastic gluten network and gluten proteins with reduced size and lower molecular weight. Yeast is also able to produce glycerol, which has a positive effect on the texture of bread, especially during freezing, and pyruvic acid [94].

#### 2.3.2. Sourdough microbial community

In sourdoughs the fermentation is spontaneous or initiated by a starter culture, by lactic acid bacteria (LAB) and yeasts fermenting a mixture of flours and water. A well-described microorganism diversity throughout the world exists, with over 30 yeast species and 50 LAB species identified. In a sourdough usually there is a dominant yeast species, usually *S. cerevisiae*, and a dominant LAB species, with low diversity, while between sourdoughs the diversity can be high. LAB cells are usually a log count higher in population compared to the yeast cells. The major metabolic activities present are acidification (LAB), flavour formation (LAB and yeasts), and leavening (yeasts and heterofermentative LAB species). The dominant yeast species in sourdoughs have the ability to ferment under both anaerobic and aerobic conditions, therefore considered Crabtree positive yeasts [98],[102].

While many papers and reviews have dealt with LAB in sourdoughs, few reviews have dealt with yeasts in sourdoughs [98],[102]. The metabolic diversity of yeast and LAB species in a sourdough offers a wide range of associations that may define the final composition and the characteristics of sourdough bread [98]. In the baking industry there is a trend to use short fermented bread-making, resulting in limited development of aroma and flavour. The addition of different bacterial starter cultures, could compensate with the production of flavour and aroma during such short fermentations [94].

#### 2.3.3. *S. cerevisiae* strains and desired characteristics

Bread is mostly made using commercial Baker's yeast. There are also commercially available sourdoughs with selected yeasts and LAB strains that offer specific and desirable characteristics depending on the targeted product. Bread though, can still be made traditionally with natural sourdough, maintained by continuous re-inoculation of new batches of flour and water [98].

Commercial strains of *S. cerevisiae* can be selected for their fermentation performance, their flavour and aroma compound production in the final product (esters, aldehydes, and ketones production), thus improving its organoleptic characteristics.

Flavour and aroma are very important parameters in bread but during bread dough fermentation, the yeast cells produce limited amount of aroma, compared to other yeast fermentations in food products. The main production of highly aromatic compounds is triggered by baking. Those compounds are either volatile like alcohols, aldehydes and ketones or nonvolatile like acids, esters, sugars, phenolic compounds free fatty acids, and lipids. The most significant of them are alcohols and aldehydes such as 2,3-butanedione and 3-hydroxy-2-butanone and esters. Some nonvolatile compounds may act as precursors for later reactions forming new flavour compounds. Sugars remaining from the fermentation react in Maillard reactions, having a great effect on aroma. The flavour is also affected with the reduction of dough pH and the production of reducing compounds which affect dough rheology [22],[94],[101].

Major importance on the strain selection is their ability to produce CO_2_ rapidly. Sucrose is preferably consumed in fermentation, so strains with strong invertase activity are preferred, but it is also necessary to select strains that are adapted to maltose utilization, especially for dough that contains little or no sucrose [22]. The ability to ferment maltose is linked to fermentation performance, because maltose is a significant source of carbon. When glucose and fructose are available, maltose-utilization enzymes are repressed causing a lag phase in CO_2_ production until the genes encoding for the maltose-utilization pathway are induced, thus in *S. cerevisiae* strain selection, the ability to ferment maltose at a high speed is a desirable feature [98]. When sucrose is added strain osmo-tolerance is to be taken into consideration [22].

Other selectable characteristics are biomass production, ethanol production, cell growth rate, dehydration, the volume of the final product, the structure, the color (carbohydrates, amino acids), cold stress-tolerance, shelf-life (acids, glycerol) [94],[98].

The strains of *S. cerevisiae* that are used in bakery are mostly polyploids. A genetic diversity study among domesticated *S. cerevisiae* strains revealed that roughly 50% of beer and bakery strains exhibited four alleles at several microsatellite loci. This suggests polyploidization and aneuploidization events in the evolution of these strains [103], a fact that was confirmed and demonstrated by a later study [104]. That study showed that some tetraploid bakery strains, reproductively isolated from *S. cerevisiae*, derived from the hybridization of different diploid *S. cerevisiae* strains, representing a new species as defined by the biological species concept [104]. An analysis of 330 bakery strains isolated worldwide showed that 75% of the commercial bakery strains and 57% of the strains isolated from natural sourdoughs are tetraploids [98].

A recent genomic analysis of 37 bakery strains showed that most strains clustered separately from the wine and sake lineages, suggesting a distinct evolutionary history, however they do not form a separate group like wine strains suggesting several different domestication courses [105].

The industries can obtain yeast cultures from culture collection centers or isolate and develop their own cultures. Maintaining the cultures long-term ensures consistency of performance and quality [95]. By using the technique enrichment culture, strains with required characteristics can be isolated from natural habitats, and selected by gradually increasing exposure to the tested factors or by cultivation with high levels of those factors over time [95]. Today, fresh yeast is generally available with the form of compressed yeast with 60–75% moisture and 44% dry content. Other forms are and dry yeast and bulk liquid or cream yeast (a suspension of fresh yeast with 82% moisture) [101]. Dried yeast can be obtained in two commercial forms: active dry yeast and instant dry yeast. Active dry yeast gives much lower leavening activity than fresh yeast, is resistant to drying, to high sugar concentration, and to some inhibitors, while instant dry yeast has a higher activity than dry yeast, approaching that of compressed yeast [95],[101]. The appropriate storage conditions for the fresh yeast to preserve the enzymatic activity and has a 15-day shelf life when stored at 4 °C. If longer storage is needed, temperature at 1 °C will suffice. Frozen yeast has a 3-month shelf-life. Dry yeasts have about 1 year (active dry yeast) or 2 years (instant active dry yeast) self-life [101].

In order to improve the characteristics of bakery products, researches study the possibility of exploiting LAB characteristics to obtain dough leavening in absence of baker's yeast, producing a ready-to-use liquid sourdough which would be added to the dough for bread Liquid sourdoughs can offer shorter, easier and more controllable procedures, properties that the industry requires [106].

Some yeast species that could be used instead of *Saccharomyces* include *Debaromyces, Kluyveromyces*, and *Schizosaccharomyces* [94]. In recent studies the use and impact of different beer yeasts on wheat bread quality, instead of Baker's yeast, was investigated, showing both superior and inferior characteristics compared to the use of Baker's yeast [107].

### 2.4. The application of *S. cerevisiae* in the chocolate industry

The beans of the tropical plant *Theobroma cacao* are the basic raw material for the production of chocolate [108]–[110]. However, raw cocoa beans are inedible, being bitter and astringent, while their aroma and flavours are not those of chocolate; thus, are subjected to fermentation to reduce the levels of polyphenols and alcaloids, causing the bitterness and astringency, and to develop flavours determining the fine organoleptics of cocoa and chocolate [108]–[110]. To this end, after the cocoa rods are opened, cocoa beans covered by the acidic [high concentration of citric acid] and sugar-rich (10–15% sugars) cocoa pulp are exposed to the naturally existing wild microflora and left to undergo a spontaneous fermentation [108]–[111].

The micro-ecosystem of the cocoa fermentation is complex and dynamic including mainly yeasts followed by lactic acid bacteria (LAB) and acetic acid bacteria [AAB] [109],[112],[113]. At the beginning of fermentation, yeast under anaerobic and low pH (3–4) conditions start to ferment the pulp-sugars producing ethanol as well as numerous flavour metabolites that will determine the quality of the final products [108]–[111],[114]. In addition, through the action of pectinolytic enzymes, they degrade gradually the highly viscous cocoa pulp (containing 1,5% pectin) allowing the air to penetrate into the pulp [108]–[111] while they also metabolise citric acid causing a pH increase [108],[110],[111] conditions that favor the growth of LAB and AAB [108]–[111]. LAB increase pH further as they metabolize citric acid and AAB oxidize ethanol to acetic acid [[108]–[111]. As both ethanol and acetic acid productions are exothermic reactions the temperature grows up to 50 °C [108]–[110].

According to experimental results, yeast activity is of paramount importance for the production of high-quality chocolate. Specifically, in pilot-scale cocoa fermentations carried out in the presence or absence of yeasts Ho et al., 2014 [115] observed that without yeasts there is a reduced production of ethanol, higher alcohols and esters throughout the fermentation while the chocolate produced was of inferior quality compared to the one produced when yeasts were present in fermentation. On the contrary, as the same research team reports LAB and AAB, were not proven necessary for the completion of cocoa fermentation, while their absence did not affect the organoleptic characteristics of the chocolate produced [116],[117].

A great variety of yeasts have been isolated and characterized from cocoa beans fermentations, with *S. cerevisiae* being among the most prevalent in several studies [112],[118],[119],[120]–[126], This fact is attributed to the specific properties of *S. cerevisiae* including its pectinolytic activity, rapid growth at a slightly increased pH and better adaptation to stress conditions of high ethanol concentrations and high temperatures [108],[109]. As unlike the other industrial fermentation, cocoa fermentation is spontaneous and consequently poorly controlled; inoculation with selected starter cultures could ensure successful fermentations with guaranteed reproducibility [108]. To this end, *S. cerevisae* has gained a lot of interest and several studies have exploited strains of the species as starters in mixed or mono-cultures fermentation schemes [127],[128]. However, the dynamics and contribution of *S. cerevisiae* to cocoa fermentation are clearly depicted in the experimental proof of the studies in which *S. cerevisiae* served as the only starter culture or in comparative studies in which its presence or absence was the only parameter of differentiation. In specific, the pectinolytic activity of *S. cerevisiae* in inoculated cocoa fermentations has been reported to improve pulp draining up to 127% [129]. In addition, Meersman et. al., 2017 [130] in order to evaluate the role of endo-polygalacturonase [EPG] conducted cocoa pulp fermentations inoculated either with a wild type *S. cerevisiae* or with a deletion strain in which *PGU1* gene, encoding EPG, was knocked out. As a control served the fermentation with non-inoculated pulp. As Meersman et al., 2017 [130] reported the viscosity of the pulp inoculated with the deletion strain wasn't significantly different from that of the corresponding non-fermented pulp whereas it was also by a 23.5% higher when compared to that of its counterpart fermented with the wild type strain [130]. These results render EPG the major pectinolytic enzyme of *S. cerevisiae* for pulp degradation.

In addition, Lefeber et al., 2012 [131] and Ramos et al., 2014 [122], have reported that the inoculation with *S. cerevisiae* strains leads to a quicker consumption of pulp-sugars and to a higher production of ethanol, accelerating the fermentation process. Several studies have also focused on the impact of *S. cerevisiae* inoculation on the organoleptic features of the chocolates produced. Lefeber et al., 2012 [131] reported that the chocolates produced in presence of *S. cerevisiae* in a mixed starter culture along with LAB and AAB were characterized as fruity and were the most preferred by a trained panel compared to their counterparts produced in its absence in the starter or through spontaneous cocoa bean fermentation. To the same conclusion resulted Visintin et al., 2017 [132] that observed more fruity odors in chocolates produced in presence of *S. cerevisiae* and *T*. *delbrueckii* than in their counterparts fermented in presence of only *T. delbrueckii*. This positive influence of *S. cerevisiae* on the sensory characteristics can be attributed to its ability to produce desirable flavour compounds such as esters, alcohols and aldehydes conferring fruity, flowery and candy, fruity and cocoa notes, respectively [133],[134]. More specifically *S. cerevisiae* has been related to key flavour compounds in cocoa beans such as ethyl octanoate, 2-phenylethyl acetate, ethyl acetate, 2-methyl-butanal, 3-methyl-butanol, 2-phenylethanol, and 2-heptanol [124],[127].

Assi-Clair et al., 2019 [135] conducted a comparative study on the performance of two *S. cerevisiae* strains when used as starters in cocoa fermentations. According to their results the two strains presented different profiles in regard to the production of flavour metabolites, depicted also on the organoleptic attributes of the produced chocolates, indicating that the selection of a successful starter is rather strain than species depending. Another parameter that must be taken into account is the cocoa variety used as the it was evidenced by the experimental results of Ramos et al., 2014 [122] and Menezes et al., 2016 [134] that inoculated different cocoa varieties with the same *S. cerevisiae* CA11 leading to chocolates with different sensory profiles, concluding that a starter culture can be appropriate for a certain cocoa variety but not for all.

One step further, tailoring of *S. cerevisiae* strains has been proposed in order to obtain superior strains with a combination of desirable features [113],[114]. First, in 2015 the Meersamn et al. [114] team mated selected strains and developed a hybrid that exhibited increased thermotolerance and fermentation capacity, compared to parental strains, whereas it also produced chocolate of superior quality. Following other mating experiments in 2016, the same team reported even improved hybrids that apart from being temperature tolerant, and robust fermentors, could also produce high concentrations of desirable esters modulating the flavour of chocolate produced [113].

Taken together, these studies underline the importance of *S. cerevisiae* in cocoa fermentations and point out to its exploitation as the starter culture to improve the efficiency and consistency of fermentations and thereafter the quality of commercial chocolate production.

## 3. The application of *S. cerevisiae* in the bioethanol industry

The history of ethanol utilization as a biofuel goes back to 1826, when the American inventor Samuel Morey designed an internal combustion engine for a boat, fueled by a mixture of ethanol and turpentine [136]. Later in 1860, Nicolaus August Otto, a German engineer, developed another internal combustion engine for which an ethanol fuel blend was used [136], followed by the American industrialist Henry Ford who constructed tractors that could be powered by ethanol [137]. The high ethanol taxes, however, as well as the much cheaper price of gasoline, prevented its use as an engine fuel. Since then, a series of historic and economic events sometimes promoted and at others opposed the idea of using ethanol as alternative energy source: the discovery of the much inexpensive petroleum, its restrictions during World Wars I and II, the oil discovery and production in the Arabic Peninsula countries and their 1970s embargo. The latter, along with the increase in fuel prizes and the environmental pollution, was a triggering event for the researchers to investigate the possibilities of alternative energy sources in a general matter. Reflecting the above expectations for inexpensive, renewable and environmentally friendly fuel, ethanol is today considered as the mostly used biofuel worldwide. The term bioethanol is used to define the amount of ethanol that is produced to be used as a fuel.

### 3.1. General aspects of the bioethanol production

Bioethanol can be used alone or mixed with gasoline and exhibits several advantages over petroleum fuel such as higher octane number (108), broader flammability limits, higher flame speeds and increased heats of vaporization [138], while on the other hand is less toxic, readily biodegradable and produces lesser air-borne pollutants [139].

Bioethanol is produced from the fermentation of sugars originating by a variety of sources, since its synthetic production is prohibitive due to its high cost. Primarily, the substrates used for sugar fermentation involve plants rich in sucrose from food crops such as sugarcane, sugar beet and a variety of fruits, and starch (corn, rice, wheat, etc): the bioethanol produced from fermentation of food crops is called ‘first generation’ biofuel. However, since these feed stocks fulfill the needs of animal and human nutrition, there is a controversy concerning their use as fermentation substrates for ethanol production. Therefore, a strategy of ‘second-generation’ biofuel has been developed, in which non-food substrates belonging to the lignocellulosic biomass (wood, straw, crop and food wastes, etc.) are exploited. Subsequently, a ‘third-generation’ bioethanol has been derived from algal biomass including microalgae and macroalgae [137],[140],[141].

United States of America use corn as the dominant feedstock for ethanol production, while Canada uses corn and wheat, Brazil sugar cane, China corn, wheat, and cassava, and European countries use primarily wheat and sugar beet to produce bioethanol [137]. Up today, USA is the largest ethanol producer worldwide for fuel utilization [142]. Ethanol production averages over a million barrels (159 million liters) per day with an annualized rate of 16 billion gallons (60 billion liters) in 2017, as reported by H. T. Kennedy in the February 17 issue of Biofuels Digest. USA and Brazil are the dominant countries in ethanol production manufacturing over 85% of the world's fuel alcohol [143].

### 3.2. Fermentation microorganisms

Alcoholic fermentation is pretty much synonymous to *Saccharomyces cerevisiae*, the protagonist in the industrial ethanol production among various other yeasts that synthesize ethanol by sugar fermentation. Under anaerobic conditions, *S. cerevisiae* uses glycolysis to catabolize sugars reaching the step of pyruvic acid formation. In follows, the latter is converted by pyruvate decarboxylase to acetaldehyde and carbon dioxide, which in turn is reduced to ethanol by alcohol dehydrogenase and releasing NAD^+^ at the same time. The terminal step reactions that lead to ethanol are therefore very important and constitute the basis for major fermentation industries [144].

Although *Saccharomyces* *cerevisiae* is the dominant sugar fermenter, other yeast species are capable of producing bioethanol from sugar fermentation as well [143]. *Kluyveromyces* *marxianus* has been investigated (among other applications) for the production of bioethanol from polyfructan substrates [145]. *Dekkera bruxellensis* has been used for bioethanol production from hexoses as products of starch hydrolysis [146], while *Scheffersomyces* (*Pichia*) *stipitis* utilizes lignocelluloses substrates [147] or algal biomass [148].

### 3.3. Contribution of *S. cerevisiae* to the synthesis of other types of biofuel

Apart from bioethanol, higher alcohols such as propanol and butanol are synthesized by genetically modified or metabolically engineered *S. cerevisiae* strains [149]. Propanol is suitable for engine fuel usage due to its high octane numbers. Since, however, its synthesis is very expensive, microbial strains have been tested for its production by fermentation of sugar substrates. Production of propanol in wine by yeast strains has been reported [150]. Based on this reaction, a genetically modified *S. cerevisiae* strain with 2-Keto acid decarboxylase (KDC) and alcohol/aldehyde dehydrogenase (ADH) activity synthesized increased amounts of propanol via 2-ketobutyrate (2KB) and could therefore be a potential for this application [151].

Butanol, as propanol, possesses a range of physical properties such as less hygroscopy, less corrosiveness, higher energy density and octane value compared with ethanol. Therefore, it can be blended with gasoline in much higher proportions than ethanol [152]. Butanol production from yeast is genetically monitored by introducing a synthetic acetone-butanol-ethanol (ABE) pathway using adh1 mutants of *S. cerevisiae* A267T/E568K, which significantly improves the n-butanol yield [153].

The isobutanol biosynthesis includes ketoisovalerate synthesis (an intermediate of valine biosynthesis) in the mitochondria and catabolism of this ketoacid into isobutanol in the cytosol [35].

A number of heterologous bacterial genes encoding appropriate enzymes which lead to the increased production of isobutanol have been introduced into *S. cereviasiae* strains. The molecular mechanisms and genetic engineering of *S. cereviasiae* strains have been analytically described and reviewed by Buijs et al., 2013 [152]. This synthesis has been applied by Butalco, Butamax and Gevoare companies that have developed commercial production of isobutanol. Butamax applied the mitochondrial pathway [154], while Butalco [155] and Gevo [156] based their production on a cytosolic pathway.

### 3.4. Characteristics of *S. cerevisiae* influencing the fermentation process

The benefits of *S. cerevisiae* as GRAS ethanologen have been extensively reviewed and include high rates of ethanol tolerance and production, stress tolerance, flexibility in genetic improvement and effective adaptation for large scale fermentations. On the other hand, *S. cerevisiae* cannot ferment certain sugars such as pentoses. Another obstacle is the accumulation of trehalose to boost the membrane as a stress response. Also, in response to osmotic stress, *S. cerevisiae* synthesizes the compatible solute glycerol (especially in the presence of high sugar concentrations) to protect the cells from water loss; this response, however, results in reduction of the proportion of alcohol production, which is undesirable in bioethanol fermentations. Subsequently, flocculation (the aggregation of yeast cells into clumps) is a phenomenon that complicates the procedure in fuel alcohol plants, because flocculent yeasts do not remain in suspension to be in contact with the fermentable sugars for the duration of the fermentation [143].

Since bioethanol is produced in fuel plants (often called biorefineries to be discriminated from the petrochemical industry), the fermentation takes place in a bioreactor, where yeast should possess tolerance in high temperature and alcohol concentration, pH alteration, and, most importantly, the achievement of the highest level of alcohol production in the shortest possible time. Therefore, because yeast is the fermentation biocatalyst, understanding yeast physiology is a key to optimizing industrial alcohol production. Various types of yeast strains have been used in fermentation for ethanol production, including wild type and recombinants obtained as hybrids, by genetic engineering, immobilization, or yeast synthetic biology approaches [141],[143].

### 3.5. The process of bioethanol production

Nutrients supporting the fermentation of certain substrates are key factors as they influence the yeast growth, stress tolerance and ethanol production along with undesired by-products. For optimal alcohol production, yeast fermentation should be supplied with appropriate nutrients which include fermentable carbohydrates, sufficient nitrogen, vitamins, as well as oxygen at the beginning of the fermentation so that yeast synthesizes compounds as e.g. sterols to strengthen its membrane [157]. Minerals are also very important, as the decarboxylation of pyruvate by yeast is catalyzed by pyruvate decarboxylase and alcohol dehydrogenase, which in turn require magnesium and zinc, respectively [144].

Regardless of the kind of feed stocks used, these should firstly undergo a pretreatment in order to reduce in size and facilitate the next steps. Usually, steam explosion is the most efficient procedure combining low cost, non-environmental hazard and complete sugar recovery [158]. The substrates should then be converted to fermentable sugars, a process carried out by enzymatic treatment which provides high selectivity for each substrate, gentle treatment and low energy cost [159]. Usually, starch is converted to hexoses by alpha amylases, while hemicelluloses are converted to pentoses by glucoamylases. Lignocellulosic material is often converted to sugars by acidic hydrolysis. Fermentation is then carried out by yeasts and ethanol is further isolated by distillation and dehydration [137],[141].

The above processes are performed by three possible mechanisms: separate hydrolysis and fermentation (SHF) followed mainly for lignocellulosic materials, simultaneous saccharification and fermentation (SSF) and simultaneous saccharification and co-fermentation (SSCF). In SSF and SSCF, enzymatic hydrolysis and fermentation process occur simultaneously and therefore are preferred for their lower cost, higher ethanol yield and shorter processing time [160],[161].

Bioethanol is produced in the bioreactor by in fed-batch, repeated batch, continuous and semicontinuous conditions [141]. In batch fermentation, where everything is added in a closed system at the beginning, the reaction manipulation is simple [162], but the high sugar concentration can act as an inhibition factor for yeast growth and therefore ethanol production [163]. In continuous fermentation, on the contrary, a bioreactor containing the fermenting yeast is constantly supplied with substrates and supporting nutrients, while the products are steadily separated from the reaction mixture [164]. The benefits include smaller volumes which lead to increased product yield and lower cost [161]. On the other hand, the long cultivation time as well as contamination risks are disadvantages for this method [165]. In fed-batch fermentation, procedures of both batch and continuous conditions are combined in order to minimize the inhibitor effects on yeast activity caused by the substrate during the batch mode by keeping it in small amounts [165]. This process exhibits many benefits and has been reported to be productive in combination with non-uniform SSF system [166].

### 3.6. Manipulation of *S. cerevisiae* to confront stress conditions in the fermentor

In a bioreactor, factors like temperature increase, pH alteration, osmotic stress and ethanol concentration can cause *S. cerevisiae* multiple stress phenomena which, in turn, will affect ethanol production. Α variation between 20–35 °C is acceptable for *S. cerevisiae* with optimum temperature growth of 30 °C, while an increase to approx. 40 °C would induce the biosynthesis of stress-response factors as heat-shock proteins and trehalose among them. The upregulation of trehalose metabolizing genes induce, in turn, a number of other genes, (for instance, those involved in ergosterol biosynthesis [167]. Subsequently, enzymes produced by *S. cerevisiae* or added externally are temperature-sensitive ant thus inactivated [168], hence temperature is monitored during the whole process. Ethanol causes toxic effects in yeast membrane, which are confronted by the addition of protective nutrients [169],[170]. On the other hand, osmotic stress can occur from the overproduction of glycerol by yeast, mainly observed in biofuel production plants that utilize starch and sugar feedstocks. This causes toxic effects on cells and decreased alcohol fermentation and could be avoided by simultaneous saccharification and fermentation [143]. SSF can be applied in fermentations of lignocelluloses feed stocks for second-generation bioethanol production as well, because during this procedure, acetic acid, which inhibits yeast growth, is reduced [171].

Non-*Saccharomyces* strains and some bacteria have been observed as contaminants in bioethanol production and compete against *Saccharomyces* starters by synthesizing inhibitory products which inhibit yeast growth and ethanol productivity. The majority of the reports come from plant industries in Brazil using sugarcane, since Brazil is the largest producer of bioethanol from this feed stock. *Dekkera*, *Schizosaccharomyces*, and *Candida* spp. are often isolated, due to their tolerance to stress conditions [172]. On the other hand, the dominant bacteria belong to the genus *Lactobacillus* and exhibit high ethanol tolerance [173]. To overcome this obstacle, the cleaning and sterilization regulation should be strictly followed in plant industries [143].

### 3.7. Strain improvement and manipulation

There is a tremendous number of *S. cerevisiae* strains used in bioethanol industry described in detail (e. g. [143],[174]). The strain requirements for an application in plant industry should include, among other parameters, stress tolerance and ethanol productivity. It has been shown that wild-type *S. cerevisiae* strains exhibit a high potential in fermenting sugars to ethanol compared to the commercial ones. This is the case of Brazilian fuel ethanol plants where wild-type *Saccharomyces* strains have been isolated from sugarcane molasses [175], or wild-type *S. cerevisiae* KL17 which reached an exceptionally high ethanol concentration of 96.9 g/L with a productivity of 3.46 g/L/h after simultaneous fermentation of glucose and galactose [176].

On the other hand, improved strains can be obtained by evolutionary adaptation, in which a strain is subjected to a particular selective stress by serial inoculations in order to obtain spontaneous mutants that responded to the above conditions [177]. By this method a series of strains possessing important properties for ethanol production have been achieved, such as xylose utilization by yeast strains used in lignocellulose fermentation [178]. Moreover, by exhibiting yeast strains xylose plus acetic acid selective pressure, Wright *et al.*, 2011 [179] isolated an improved strain fermenting xylose and resistant to acetic acid and proceeded thus furthermore in strain improvement to be used in second-generation ethanol fermentation.

In addition, enhanced strains for certain properties can be developed by application of classical genetics, such as hybridization by crossing or protoplast fusion, as well as mutagenesis. These methods have been extensively studied by Steensels et al., 2014 [174]. For instance, hybrid strains developed by protoplast fusion between *S. cerevisiae* and non-*Saccharomyces* strains which ferment xylose have been used for fermentation of *Ipomea carnea* biomass containing both hexoses and pentoses [180]. By hybrid strategies, appropriate strains have been developed (especially in ethanol tolerance) with natural method and therefore are not regarded as genetically modified (GM). On the other hand, many industrial strains are polyploid or aneuploid and therefore cannot be improved by mating procedures. By the crossing methods, many other characteristics apart from the desired are altered and could affect the fermentation procedure.

Cell immobilization is a common technology followed in fermentation procedures. It exhibits a series of advantages over free cell fermentation such as high density of active cells, higher rates of conversion, while the reaction time is shortened and the product isolation is facilitated. The immobilization types include adsorption, crosslinking, encapsulation and entrapment. The most popular immobilization method for yeast is adsorption, while calcium alginate has been the most suitable carrier. A number of immobilization procedures concerning strains, carriers and ethanol productivity have been reviewed by Azhar et al., 2017 [141]. Among them, a high ethanol production of 98.48 g/L has been achieved by fermentation of sweet sorghum juice from *S. cerevisiae* NP 01 immobilized by adsorption on sorghum stalk [181].

There is a remarkable variety of genetic modified yeast strains used in alcohol fuel plants, in which heterologous enzymes providing products of high added value have been introduced. Recombinant DNA technology is applied depending of the desired properties that should be provided by the recombinant strains. The reduction of glycerol production has been a challenge achieved for ethanol-producing yeasts [182]. For the fermentation of starch obtained from corn mashes (mainly in USA), commercial strains modified to express glucoamylase genes have been constructed. This way, starch is breaking down to glucose, which, in turn, is fermented in a SSF system, where glucose is slowly released and the osmotic stress is diminished [183]. Another obstacle confronted by GM yeasts is the release of pentoses after the pretreatment of lignocellulosic substrate, not fermented by wild *S. cerevisiae* strains. The introduction of heterologous xylose isomerase genes in wild type strains allows the complete utilization of lignocellulosic hydrolysates [184]. Thus, the development of robust yeast strains with improved metabolic pathways will continue to be critical for the fruitful operation of large-scale ethanol biorefineries.

## 4. Conclusions

*S*. *cerevisiae* is undeniably the best studied and one of the most widely used eukaryotes in a wide variety of industrial processes, such as wine, food and ethanol production. Despite of the efficient adaptation of the various *S*. *cerevisiae* strains used in those processes, there is still a great potential of either optimizing existing strains, or exploit the immense natural reservoir of environmental isolates.
